# The Impact of Consumer Purchase Behavior Changes on the Business Model Design of Consumer Services Companies Over the Course of COVID-19

**DOI:** 10.3389/fpsyg.2022.818845

**Published:** 2022-03-03

**Authors:** Hu Tao, Xin Sun, Xia Liu, Jinfang Tian, Di Zhang

**Affiliations:** ^1^School of Business and Administration, Shandong University of Finance and Economics, Jinan, China; ^2^School of Statistics, Shandong University of Finance and Economics, Jinan, China

**Keywords:** consumer psychology, consumer purchase behavior, efficiency-centered business model, novelty-centered business model, consumer services company

## Abstract

The COVID-19 pandemic has had a profound psychological and behavioral impact on people around the world. Consumer purchase behaviors have thus changed greatly, and consumer services companies need to adjust their business models to adapt to this change. From the perspective of consumer psychology, this paper explores the impact of consumer purchase behavior changes over the course of the pandemic on the business model design of consumer services companies using a representative survey of 1,742 individuals. Our results show that changes in consumer purchase behavior have a significant impact on the design of consumer services firms’ business models. Specifically, changes in consumers’ purchase object, motive, and timeframe are more likely to spark a novelty-centered business model design, whereas changes in purchase method tend to inspire an efficiency-centered one. Our findings provide a theoretical reference for consumer services companies in designing business models when faced with unexpected crises.

## Introduction

The COVID-19 outbreak has abruptly disrupted the global political and economic order (Fernandes, 2020), significantly impacting consumer services sectors such as retailing, hospitality, and tourism (Pantano et al., 2020). The pandemic has resulted in unprecedentedly large-scale lockdowns across the world (Kuckertz et al., 2020), severely restricting people’s daily activities. As a result, more consumer services companies are experimenting with new technologies and platforms in order to meet the changing consumer demands, leading to new consumption patterns. To cope with the restrictions, some consumer services companies have developed alternative business models, such as “contactless delivery” and “social cinema.”

The government’s strict restriction on population movement has led to seismic shifts in people’s livelihoods and daily lives. More people are suffering from depression and loneliness, and some have resorted to alcohol, drugs, or even self-harm for relief (Alsukah et al., 2020). These unhealthy emotions and behaviors have caused quite shifts in individuals’ consumption psychology: people in a dire circumstance may develop a “nothing to lose” mentality and become more prone to risk-taking, resulting in more impulse purchases (Hill et al., 1997; Harris et al., 2002); they might also develop post-traumatic stress disorder (PTSD) and future anxiety, resulting in fewer purchases to increase savings (Nolen-Hoeksema and Morrow, 1991; Kılıç and Ulusoy, 2003; Kun et al., 2013). During the COVID-19 pandemic, consumer psychology and purchase behavior have fundamentally changed.

Purchase behavior is a special and specific behavior that directly reflects people’s needs, desires, pursuit of material and spiritual interests (Braithwaite and Scott, 1990). Factors that affect changes in purchase behavior include social factors, cultural factors, demographic factors, and situational factors (Cici and Bilginer Özsaatcı, 2021). Therefore, the COVID-19 pandemic as a social factor is also affecting different changes in purchase behavior. Scholars generally believe that a large number of consumers showed panic buying behavior or impulsive buying behavior in the early stage of the COVID-19 pandemic (Aljanabi, 2021; Stuart et al., 2021), and even accompanied by compulsive buying behavior (Samet and Gözde, 2021). While purchase behavior in the middle of the COVID-19 pandemic is characterized by mobility (Gao et al., 2020; Zhang et al., 2020; Lu et al., 2021). The application of digital technology has created favorable conditions for consumers to participate in online shopping, and consumers’ online purchase activities have increased significantly (Jiang and Nikolaos, 2021). However, the changes in purchase behavior in the above literature focus on changes in a single dimension, and do not systematically sort out the changes in consumer purchase behavior under the COVID-19 pandemic. Therefore, according to the basic theory of marketing, this study systematically sorts out the multiple dimensions of changes in consumer purchase behavior under the COVID-19 pandemic, and improves the items of the purchase behavior changes in each dimension, so as to provide supplements for the theory of consumer behavior.

Countries around the world have adopted special measures such as regional blockades in the process of fighting the epidemic. These measures are a shock to traditional business models and require corresponding changes to traditional business models. However, there are currently different perspectives on the impact of purchase behavior on corporate marketing models, including traditional brick-and-mortar store purchase models, green marketing models, B2B transaction models, and online marketing models (Beuckels and Hudders, 2016; Nguyen et al., 2016; Sundström et al., 2019; Wei and Ho, 2019). However, there is little literature analyzing the impact of purchase behavior on firms’ business models from the perspective of sudden crisis events. Besides, there are many external factors affecting business model design, such as technological change (Øiestad and Bugge, 2014), contextual factors (Zott and Amit, 2013; Ghezzi et al., 2015), local market opportunities (Sinkovics et al., 2014), and third-party partnerships in the customer value proposition development (Velu, 2015). Among the above-mentioned external factors affecting business model innovation, less research is based on the impact of residents’ behavior. Therefore, it is particularly important to study the impact of changes in consumer purchase behavior on business model design in the context of the COVID-19 pandemic.

To answer these questions, this paper examines consumers’ psychological changes over the course of the COVID-19 pandemic based on the theory of environmental psychology, affective psychology, and consumer psychology. The stimulus-organism- response (S-O-R) model (Mehrabian and Russell, 1974) is used to explain how the pandemic triggered people’s psychological alteration, which in turn sparked changes in their purchase behavior. Then, we conduct a representative survey of 1742 individuals to explore the impact of customer purchase behavior changes on the business model design of consumer services companies using the expectation confirmation theoretical model (Oliver, 1980). The remainder of this article is structured as follows: Section 2 is devoted to conceptual basis and research assumptions; Section 3 presents the research design; Section 4 is the empirical analysis; Section 5 concludes the paper.

## Conceptual Basis and Research Assumptions

### Consumer Purchase Behavior Changes During the COVID-19 Pandemic

According to disaster psychology, different psychological changes of residents caused by different periods of emergencies make purchasing behaviors show distinctive characteristics, such as panic buying behaviors, impulse buying behaviors, compulsive buying behaviors and online buying behaviors. In the initial stage of the COVID-19 outbreak, although only the individuals who experienced the event will be directly affected, the negative emotions caused will be transmitted to the entire society through social networks. The public is prone to irrational emotions, including anxiety and depression (Clauw et al., 2003; Klitzman and Freudenberg, 2003). Public anxiety, especially in the face of a large-scale pandemic, can easily lead to the spread of negative emotions (Hull et al., 2003). In addition, consumers’ perception of uncertainty, scarcity, and severity and other psychological factors will increase, causing customers to panic buying behavior (Omar et al., 2021). The specific performance is to stock up on some necessities and reduce the purchase of non-essential items (Roşu et al., 2021). The fear of out-of-stocks and supply chain disruptions brought about by the COVID-19 pandemic will also increase consumers’ impulse buying behavior. The worse consumers perceive the COVID-19 outbreak, the stronger their inner fears, and the more likely they will lead to their impulsive purchases of health products. The COVID-19 pandemic has increased the perceived pressure of consumers, and some consumers are accompanied by compulsive purchasing behavior. By increasing their buying behaviors, they can relieve their inner anxiety and tension (Samet and Gözde, 2021). Besides, online purchasing behaviors have become increasingly popular with consumers after the COVID-19 outbreak. In the face of the government’s home isolation measures, it has become more and more common for consumers to use online shopping for food and other items. People who are aware of the risks of going out are more willing to buy fresh food online (Lu et al., 2021). Consumer purchase behavior is no longer limited by time and space, and consumers use mobile tools such as mobile phones to achieve shopping freedom (Zhang et al., 2020). Among the above-mentioned studies have carried out detailed research on a certain characteristic of changes in consumer purchase behavior, but have not systematically sorted out changes of the psychological characteristics and behavioral characteristics of consumers. Changes in consumer purchase behavior are reflected in many aspects, not just a single dimension of change.

The stimulus-organism-response (S-O-R) model reveals the influence of the environment on individual emotions. “Stimulus” refers to any environmental factor that causes an individual’s intrinsic response to the environment. “Organism” represents the individual’s emotional state and cognitive process (Zinkhan et al., 1992). “Response” is the individual’s response to the external stimulus (Hunt and Downing, 1990). In short, the S-O-R theory states that external stimulus triggers people’s emotional and cognitive changes, which in turn lead to different behaviors. Therefore, the COVID-19 pandemic as the external stimulus will change people’s consumption psychology and hence their purchase behavior in terms of purchase object, motive, place, timeframe, and method.

In terms of purchase object, the outbreak of the epidemic has made consumers put forward higher requirements for products or services. When consumers face an emergency, they choose problem-solving products or services over emotional healing products or services (Yeung and Fung, 2007; Cai et al., 2020). Utilitarian products, as opposed to hedonic items, are more effective in addressing consumers’ immediate needs (Yang et al., 2020). Consumers caught in the pandemic would increase their purchases of utilitarian products such as disinfectants, masks, and health foods. On the other hand, when people are under pressure or are anxious about external threats, instead of directly addressing the issues, they often activate a psychological defense mechanism—the cognitive and behavioral tendencies that individuals unconsciously adopt in the face of frustration or conflict in order to relieve tension and anxiety (Cramer, 1991)—to protect themselves (Baumeister et al., 1998). The COVID-19 pandemic has triggered people’s psychological defense mechanism, leading to more cautious buying. Consumers are not only more price-sensitive, but they also demand higher-quality and more reliable products. In terms of purchase objects, consumers pay more attention to the quality of the objects they buy. The increase in online purchasing activities has also made consumers more willing to disclose their personal information (Gao et al., 2020).

In terms of purchase motive, previous scholars can divide purchase motivation into hedonic motivation, social motivation and utilitarian motivation (Voss et al., 2003). This framework, which shapes consumer motivation for product categories, has been widely used in the field of consumer behavior. In recent years, the application of new technologies has become more and more extensive. Therefore, new media is used by more and more people and brings more fun to consumers. Driven by hedonic motivation, consumers are more keen on new media shopping methods such as Douyin and Kuaishou (Koch et al., 2020). The contribution of social responsibility can improve consumers’ willingness to purchase in advance (Tong et al., 2021). During the COVID-19 pandemic, many Chinese companies have donated financial and material resources during the pandemic, which helped build positive customer perceptions and attitudes toward their products (Yin et al., 2019). Therefore, driven by social motivation, consumers are more willing to choose brands that have contributed to society. In addition, consumers’ herd mentality makes them more utilitarian in the process of purchasing goods, and thus more willing to choose products with higher evaluation (Samet and Gözde, 2021). Driven by the above motivation, consumers choose more and more brands of goods.

In terms of purchase place, the government’s home isolation measures have made consumers’ offline shopping channels difficult, and their online purchases have become more and more common (Zhang et al., 2020; Lu et al., 2021). Specifically, consumers have gradually developed the habit of purchasing some daily necessities online. At the same time, the rapid development of social media has brought more shopping convenience to consumers. As a result, when consumers shop on social platforms such as WeChat (Larios-Gómez et al., 2021), they are able to pick their favorite products more quickly (Ali et al., 2021). As the number of consumers on social platforms increases, the number of consumers in offline venues decreases accordingly. Although consumers’ offline purchasing activities have decreased, consumers have become more demanding of offline shopping places. In order to reduce the risk of infection, when consumers shop offline, they pay more attention to the safety, convenience and goodwill of shopping places (Butu et al., 2020). As a result, consumers have also changed significantly in terms of purchase place.

When it comes to purchase timeframe, advances in technology stimulate consumers’ perception of the value of time. The new shopping habits that consumers have formed during the COVID-19 epidemic have made their sense of time sharper than before the COVID-19 outbreak. Consumers expect the fastest way to obtain goods and services (Kyowon et al., 2020), improving their shopping efficiency. The development of Internet technology and the wide application of mobile terminals have enabled consumers to satisfy their desire to shop anytime, anywhere. Therefore, consumers prefer a shopping method with unlimited time to purchase goods and less time-consuming in terms of purchase timeframe.

In terms of purchase method, in order to avoid contact with uncertain external services and reduce the risk of infection, consumers choose contactless delivery methods based on safety needs (Larios-Gómez et al., 2021). Through the contactless delivery method, consumers can effectively relieve their inner anxiety and smoothly maintain the order of daily life.

### Consumer Purchase Behavior Changes and Business Model Design

People’s fear and anxiety about the pandemic are unlikely to abate in the near future, and the resulting changes in consumer demand might eventually damage the supply chain performance of consumer services companies (Ivanov, 2020). These companies have already been experiencing significant challenges with their existing business models due to strict social isolation, delayed return-to-work, and disrupted logistics. The pandemic is putting some major businesses to the test since consumers may not restore their previous buying habits anytime soon (Pantano et al., 2020). According to the Expectation Confirmation Theory, consumer services companies have to adjust their business models to meet new customer expectations in order to obtain consumer satisfaction.

Changes in consumer purchase behavior under the COVID-19 pandemic have had an impact on the design of novelty-centered business models. Novelty-centered business models place more emphasis on exploiting new opportunities in new ways (Foss and Saebi, 2017), and their essence is to satisfy new customer value propositions, need or experience through innovations in the content, structure or governance of the activity system. Although the COVID-19 pandemic has led to a decline in consumers’ purchase power, the requirements for product quality upgrades will not change. Changes in purchase object drives consumer services companies to design novelty-centered business models. With the improvement of consumers’ overall consumption level, the enhancement of consumption power and the upgrade of consumption preferences, their satisfaction with standardized products gradually decreases, and the trend of pursuing more diversified and personalized products or services will continue. As consumer preferences increase in diversification, companies must launch new products and price them appropriately in the face of a fiercely competitive market, especially in the context of environmental uncertainty exacerbated by the COVID-19 pandemic. Novelty-centered business models can bring customers better products and experience through innovative methods on the basis of product technology innovation.

When it comes to purchase motive, consumers prefer products from companies with a reputable image or a strong sense of social responsibility. Branded products increase consumers’ perceived usefulness (Bhattacherjee, 2001), which is precisely what novelty-centered business models could accomplish. Therefore, consumers expect companies to design novelty-centered business models. In terms of purchase method, consumers prefer novel purchase methods and services such as mobile payment and contactless delivery. This suggests that consumer demand for novel payment methods has not yet been completely satisfied. People who get exposed to the same products or services repeatedly will eventually get bored due to the diminishing marginal utility of overexposure (Line et al., 2016). Bored customers will eventually feel less satisfied. Thus, consumer services companies should adopt a novelty-centered business model design in order to re-establish customer satisfaction. Moreover, consumers tend to favor a shorter purchase timeframe and a safer purchase place, indicating their expectation to reduce perceived risks (Garaus and Garaus, 2021). To meet that expectation, firms would be better served by novelty-centered business model design. Therefore, changes in consumer purchase behavior have led to the emergence of novelty-centered business models. In summary, the following assumption is made:

H1a: Changes in purchase object facilitate the design of novelty-centered business models.
H1b: Changes in purchase motive facilitate the design of novelty-centered business models.
H1c: Changes in purchase place facilitate the design of novelty-centered business models.
H1d: Changes in purchase timeframe facilitate the design of novelty-centered business models.
H1e: Changes in purchase method facilitate the design of novelty-centered business models.

Changes in consumer purchase behavior under the COVID-19 pandemic have had an impact on the design of efficiency-centered business models. Consumer purchase behavior is a process from information acquisition, formation of purchase intention to purchase decision-making problem. Consumer purchase intention is an important factor that determines the final purchase decision. And information is an important factor that affects consumers’ purchasing intention and ultimately making purchasing decisions. Generally speaking, consumers are risk-averse, so they will collect a lot of relevant information before purchasing, so as to turn the uncertainty of purchasing a certain product into certainty. With the rapid development of information technology, whether the contradiction between the explosive growth of information and the limited attention of consumers can be resolved has become an inevitable requirement for enterprises to gain a competitive advantage. The rapid development of information technology also brings the risk of personal information being infringed on consumers at all times in the transaction, especially in the field of online consumption, the black industry chain of “stealing” and “illegal use” of consumers’ personal information shows an explosive growth trend. Whether companies can keep the personal information of consumers collected in business activities strictly confidential has become a matter of close concern to consumers. Efficiency-centered business models emphasize that enterprises can improve business efficiency by reducing transaction costs, improving information transparency and sharing, and improving transaction security. With this, information can be efficiently shared between customers and enterprises, and the “information island” between the two can be reduced, so that consumers can trust enterprises and generate purchase intentions.

In terms of purchase object, people are more rational in choosing what to purchase. This increases consumer demand for efficiency in the products or services purchased from consumer services companies. In this case, companies should choose an efficiency-centered business model design since it emphasizes improving the efficiency of business transactions. Customers are satisfied, and their expectations are confirmed when they perceive that the efficiency of the goods or services exceeds the expected efficiency. In addition, with regards to purchase motive, consumers tend to favor brands that are well rated and contribute to society. Consumers perceive branded products as allowing them to make the right choice more quickly. Efficiency-centered business models are consistent with this consumer perception. In terms of purchase place, people prefer to shop online or on social media platforms, highlighting their expectations for a safe and convenient shopping environment. Efficiency-centered business models are essential for firms to meet such customer expectations. As mentioned above, consumers prefer a shorter purchase timeframe, indicating that consumers’ time efficiency expectations have not been fully satisfied and the increasing need for consumer services companies to develop efficiency-centered business models. In terms of purchase method, the fact that consumers have become more favorable in mobile payment and contactless delivery reflects the growing consumer demand for efficient payment and delivery methods. Hence, consumer services companies need to design an efficiency-centered business model in order to increase customer satisfaction. Therefore, in addition to novelty-centered business models, the change in consumer purchase behavior has also created a demand for efficiency-centered business models. In summary, the following assumption is made:

H2a: Changes in purchase object facilitate the design of efficiency-centered business models.
H2b: Changes in purchase motive facilitate the design of efficiency-centered business models.
H2c: Changes in purchase place facilitate the design of efficiency-centered business models.
H2d: Changes in purchase timeframe facilitate the design of efficiency-centered business models.
H2e: Changes in purchase method facilitate the design of efficiency-centered business models.

**FIGURE 1:**
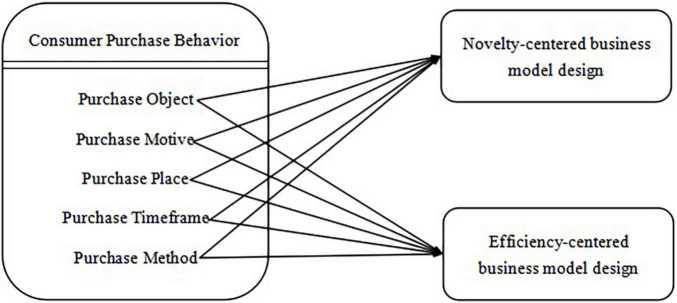
Conceptual model.

On the basis of drawing on relevant research and theoretical achievements, this research innovatively constructs a theoretical research model of consumer purchase behavior on business model innovation under the background of normalization of the epidemic (Figure 1).

## Research Design

### Survey Design and Variable Measurements

The data used in this paper was obtained through a representative survey. In order to ensure the reliability of the questionnaire, the design of the changes in consumer purchase behavior questionnaire adopted the literature method to select the measurement variables and corresponding items of the related research on consumer purchase behavior. On this basis, researchers related to consumer behavior were invited to evaluate the questionnaire, and potential consumers were selected as the survey objects for interviews, and some difficult and ambiguous questions were revised and supplemented. The scales for business model design mainly refer to the mature scales of relevant international studies. Then, modifications were made to account for the unique circumstances of the COVID-19 pandemic. On this basis, 58 individuals were selected for pre-investigation. Based on the test results, questions with relatively low factor loadings were further revised. Then, a rating scale is developed. The questionnaire take a form of a five-point Likert scale, where 1 = *Strongly disagree*, 2 = *Disagree*, 3 = *Not sure*, 4 = *Agree*, and 5 = *Strongly agree*. The main variables include basic demographic characteristics, the changes in consumer purchase behavior (purchase object, motive, place, timeframe, and method), and business model designs (novelty- and efficiency-centered).

#### Dependent Variable

Business model design (BMD) was chosen as the dependent variable. Based on Zott and Amit (2009), we categorized business model design into novelty-centered business model design (NBM) and efficiency-centered business model design (EBM). The survey questionnaire was similar to that provided by Zott and Amit, with a few modifications to account for the COVID-19 pandemic. NBM was measured by ten items: (1) ‘*The merchant offers a wider range of goods to attract new customers*’; (2) ‘*The merchant offers a wider range of services to attract new customers*’; (3) ‘*The merchant offers a broader selection of brands*’; (4) ‘*The merchant is using more of a combination of physical and online shops to offer goods or service*’; (5) ‘*The merchant has adopted a wider variety of payment methods*’; (6) ‘*The merchant has become an industry benchmark*’; (7) ‘*The merchant is more creative in its stor*e design’; (8) ‘*The merchant offers more innovative products*’; (9) ‘*The merchant offers more innovative services*’; (10) ‘*The merchant’s business model is new*’. EBM was measured by eight items: (1) ‘*The merchant has made my purchase of goods or services more efficient*’; (2) *The merchant made my shopping time shorter*’; (3) ‘*The merchant has given me more information about the goods*’; (4) ‘*The merchant has given me more information about the services*’; (5) ‘T*he merchant gave me more ways to buy and settle my bill*’; (6) ‘*The merchant made fewer errors in the sales process*’; (7) ‘*The merchant offers cheaper goods or services*’; (8) ‘*My communication with the merchant is faster and more efficient.*’

#### Explanatory Variable

Consumer purchase behavior changes (CPC) were the explanatory variable. Based on Valaskova et al. (2021) and Vázquez-Martínez et al. (2021), we described consumer purchase behavior changes from five dimensions: changes in purchase object (PO), changes in purchase motive (PR), changes in purchase place (PP), changes in purchase timeframe (PT), and changes in purchase method (PW). As before, we made a few modifications to the questions measuring these variables to account for the COVID-19 pandemic. The specific measurements of each dimension were as follows.

According to marketing theory, changes in purchase object refers to the goods or services that consumers buy. Based on Zhang and Zheng (2019), Consumers’ choice of purchase object is mainly reflected in price, quality and service. The measurement of the purchase object is measured from the above three aspects. At the same time, combining the characteristics of purchasing behavior under the COVID-19 pandemic (Cai et al., 2020; Gao et al., 2020; Yang et al., 2020) and the results of interviews with consumers, the changes in purchase object (PO) were measured by nine items. As follows: (1) ‘*I am more likely to buy technology products (e.g., sports bracelets, etc.)*’; (2) ‘*I am more likely to buy high protein products (e.g., milk, etc.)*’; (3) ‘*I am more likely to buy high-end products*’; (4) ‘*I am more likely to buy personalized items’; (5) ‘I am more cautious about buying non-essential products*’; (6) ‘*I have higher expectations of customer service for the products I buy*’; (7) ‘*I am more concerned about the quality and efficacy of products*’; (8) ‘*I am more concerned about the price of products*’; (9) ‘*I am more likely to allow merchants access to my personal information.*’

The hedonic shopping motivation research scale according to Mark and Kristy (2003) and the utilitarian shopping motivation research scale by Martínez-López et al. (2014) formed the basis of the measurement scale of change in purchasing motivation in this study. On this basis, items unrelated to the COVID-19 pandemic were eliminated, and some items were improved to form a new measurement scale. The changes in purchase motive (PR) were measured by five items: (1) ‘*I am more likely to buy highly rated products*’; (2) ‘*I am more likely to try new brands*’; (3) ‘*I am more likely to buy products recommended by acquaintances*’; (4) ‘*I am more likely to buy products recommended in short video apps such as Douyin (Chinese TikTok) and Kuaishou*’; (5) ‘*I prefer brands that have contributed to society during the COVID-19 pandemic.*’

Marketing practice differentiates the place of purchase into online and offline (Srikanth et al., 2011). Based on Volpe et al. (2013), we measured the change in offline purchase location in purchase place. Ali et al. (2021) and Larios-Gómez et al. (2021) provided us with measurement items for changes in online purchase place. The changes in purchase place (PP) were measured by five items: (1) ‘*I am more likely to shop in a one-stop store*’; (2) ‘*I am more likely to buy goods in a contactless store*’; (3) ‘*I am more concerned about the safety of the shopping environment*’; (4) ‘*I am more concerned about the reputation of merchants*’; (5) ‘*I am more willing to shop on social media platforms such as WeChat.*’

Based on Eastlick and Feinberg (1999), Consumers’ requirements for purchase timeframe were reflected in flexibility, speed and convenience. The survey questionnaire was similar to that provided by Eastlick, with a few modifications to account for the COVID-19 pandemic. Combined the results of the interviews, the changes in purchase timeframe (PT) were measured by three items. As follows: (1) ‘*I am more likely to spend an unlimited amount of time shopping*’; (2) ‘*I am more likely to spend less time shopping*’; (3) ‘*I am more organized in my shopping activities, such as making detailed shopping lists, planning shopping routes, and so forth.*’

Finally, based on Larios-Gómez et al. (2021) and the results of interviews with consumers, the changes in purchase method (PW) were measured by three items: (1) ‘*I am more willing to accept contactless delivery services*’; (2) ‘*I am more willing to use mobile payment*’; (3) ‘*I am more willing to use self-checkout.*’

#### Control Variable

Following existing literature, we selected respondents’ gender (Gender), age (Age), education attainment (Edu), and monthly income level (Income) as control variables.

To ensure measurement precision and accuracy, the data were analyzed using the item response theory (IRT) model rather than factor analysis, as the latter results in information loss (Xue et al., 2019). The Item Response Theory (IRT) model estimates variables through an iterative computation process, making sufficient use of existing information. The IRT model also takes into account the difficulties of survey questions, making the estimations closer to real practice (Xue et al., 2021). Therefore, we utilized the IRT model to measure business model design (BMD), including novelty-centered business model design (NBM) and efficiency-centered business model design (EBM).

Rabe-Hesketh et al. (2004) propose two types of IRT model, i.e., one-parameter logistic IRT (1PL-IRT) model and two-parameter logistic IRT (2PL-IRT) model. However, it is unrealistic to apply the 1PL-IRT model in real practices. Therefore, the 2PL-IRT model is widely used to measure latent variables. Given the fact that the 2PL-IRT model can only be applied to estimate binary variables, Zheng and Rabe-Hesketh (2007) integrate the partial credit model (PCM) into the 2PL-IRT model, namely the 2PL-PCM, to measure latent variables with multiple categories. Therefore, following Xue et al. (2019), we employed the 2PL-PCM to measure BMD and NBM. The 2PL-PCM model specifications are as follows.


(1) $$\mathrm{Pr}\left(x_{i\,n}=j|\theta_{n}\right)=\frac{\exp\left\{\sum_{m=2}^{j}\gamma_{i}\,\left(\theta_{n}-\delta_{i\,m}\right)\right\}}{1+\sum_{l=2}^{k_{i}}\exp\left\{\sum_{m=2}^{l}\gamma_{i}\,\left(\theta_{n}-\delta_{i\,m}\right)\right\}}$$

(2) $$\ln\frac{\mathrm{Pr}\left(x_{i\,n}=j|\theta_{n}\right)}{\mathrm{Pr}\left(x_{i\,n}=j-1|\theta_{n}\right)}=\gamma_{i}\,\left(\theta_{n}-\delta_{i\,j}\right)$$

### Data

This paper aims to investigate the impact of consumer purchase behavior changes on the business model design of consumer services companies during the COVID-19 pandemic. The intended population for this research was identified as individuals who have shopped during the COVID-19 pandemic and have a basic understanding of consumer services business models. We fielded the survey from 18 April 2020 to 23 July 2020. All questionnaires were anonymous, and rigorous distribution and return protocols were followed. Questionnaires were distributed in three main ways: first, upon contact confirmation, our team members conducted on-site interviews with the respondents and distributed the questionnaires; second, using the team members’ social connections, the questionnaires were distributed to those who qualified; Third, the questionnaires were distributed through email. In the end, a total of 1,887 questionnaires were distributed, and 1,742 were valid following careful screening.

The demographic profile of the respondents is as follows. Male respondents account for 43.456%, while female respondents account for 56.544%. In terms of age, 0.459% of the respondents are under the age of 18; 30.540% are between 18 and 25 years old; 25.316% are between 26 and 35 years old; 19.518% are between 36 and 45 years old; 19.346% are between 46 and 55 years old; 4.822% are 56 years and above. Regarding education attainment, 1.607% of the respondents have a junior secondary certificate or below; 6.257% have a senior secondary certificate (including high school and vocational and technical school certificate); 48.565% have a university certificate; 43.571% have a postgraduate certificate or above. Finally, in respect of monthly income level, 19.460% of the respondents earn no income; 6.889% earn less than RMB 2,000 per month; 18.657% earn RMB 2,001–5,000 per month; 24.799% earn RMB 5,001–8,000 per month; 30.195% earn RMB 8,001 or more per month.

Common method variance (CMV) is likely to lead to biased results for variables obtained from survey questionnaires (Xue et al., 2019). Therefore, we employed the Harman’s single factor test to examine the existence of the CMV. The test results showed that the common factor only explains 18.733% of total variance, indicating that the common method bias is not a concern for this paper.

## Empirical Analysis

This section presents the empirical analysis conducted on the collected survey questionnaires. It includes four parts: (1) descriptive statistical analysis and correlation coefficient analysis; (2) analysis of consumer purchase behavior changes by demographic characteristics (including gender, age, monthly income level, and education attainment); (3) regression modeling; (4) robustness tests.

### Descriptive Statistical Analysis and Correlation Coefficient Analysis

Table 1 showed the descriptive statistics of the main variables. All variables have a relatively small mean value, indicating that respondents’ willingness to change their behavior for the pandemic is low. A plausible explanation is that people became less vigilant and concerned as the pandemic was gradually brought under control. On the other hand, the novelty-centered business model has a higher mean value than the efficiency-centered business model, suggesting that following the pandemic, respondents tend to favor the novelty-centered business model over the efficiency-centered one. This is because as the outbreak gradually subsides, people become less pessimistic and hence more interested in new things. In addition, the standard deviations of all variables are small, indicating small variations for variables used in this study. This is also reflected in the extreme deviations, with the largest extreme deviation being only 5. Moreover, all the variables range from −3 to 2, indicating no extreme values observed.

**TABLE 1:** Descriptive statistics and correlation coefficients.

	Obs.	Mean	SD	Min.	Max.	NBM	EBM	PO	PR	PP	PT	PW
*NBM*	1742	0.170	0.865	−2.000	2.000	1.000						
*EBM*	1742	−0.160	0.862	−3.000	1.000	0.807**	1.000					
*PO*	1742	−0.020	0.842	−3.000	2.000	0.562**	0.504**	1.000				
*PR*	1742	−0.030	0.749	−3.000	2.000	0.509**	0.433**	0.659**	1.000			
*PP*	1742	−0.050	0.801	−3.000	1.000	0.469**	0.468**	0.626**	0.565**	1.000		
*PT*	1742	−0.060	0.780	−2.000	1.000	0.452**	0.442**	0.592**	0.430**	0.566**	1.000	
*PW*	1742	−0.080	0.792	−2.000	1.000	0.418**	0.455**	0.434**	0.400**	0.500**	0.481**	1.000

***, **, * represent significant at the 1, 5, and 10% significant level, respectively; T-values are provided in parentheses.

Table 1 also showed the correlation coefficients between the main variables. The results indicate a significant and positive correlation between consumer purchase behavior changes and both types of business model designs. However, the correlation between consumer purchase behavior changes and novelty-centered business model design is more significant; the impact of consumer purchasing behavior changes on novelty-centered business model designs is likely to be greater. However, the exact relationships between the variables remain to be tested further below.

### Analysis of Consumer Purchase Behavior Changes by Demographic Characteristics

**FIGURE 2:**
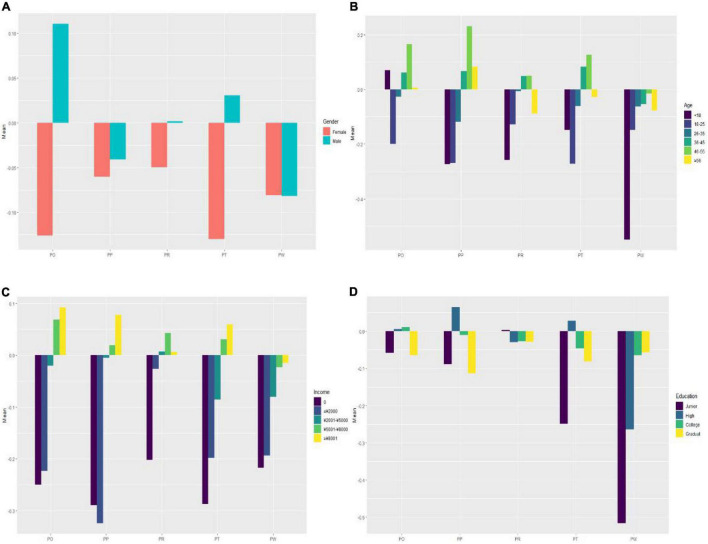
Consumer purchase behavior changes by demographic characteristics. **(A)** Consumer purchase behavior changes by gender. **(B)** Consumer purchase behavior changes by age. **(C)** Consumer purchase behavior changes by monthly income level. **(D)** Consumer purchase behavior changes by education attainment.

Over the course of the COVID-19 pandemic, consumer purchase behaviors have changed dramatically. These changes exhibited a number of differences according to demographic characteristics. Figure 2 illustrated the differences in consumer purchase behavior changes by gender, age, monthly income level, and education attainment. Details were be discussed in the following four sub-sections.

#### Gender

Figure 2A displayed the pandemic-induced changes in consumer purchase behavior by gender. The changes in purchase object and timeframe exhibited an apparent gender variation. Females usually tend to act more impulsively than males (Hesham et al., 2021). The pandemic have prompted male consumers to be more rational in shopping; therefore, the purchase behavior change of male consumers is greater. In terms of purchase place and motive, a relatively small gender variation is shown. Lastly, no significant gender variation is found for changes in purchase method.

#### Age

Figure 2B showed the pandemic-induced change in consumer purchase behavior by age group. Observably, all parameters of consumer purchase behavior changed exhibit age variation. Individuals aged 18–25 and 26–35 showed a smaller change in purchase object, but those aged under 18, 36–45, 46–55, and 56+ showed the opposite. The change in purchase place followed a similar pattern, with the exception that persons aged under 18 exhibited a lesser change. In terms of purchase motive and timeframe, the variation across age groups was minor; individuals aged 36–45 and 46–55 showed a greater change while other age groups showed less change. Lastly, the change in purchase method was relatively small across all age groups. The reason for that is: young people had already adapted to the online lifestyle before the COVID-19 outbreak, therefore no significant change after; but for the elderly, although they tend to be more skeptical of the internet, they now have little choice but to purchase online due to the pandemic isolation and lockdown. Overall, the middle-aged and elderly have changed the most in their purchase behavior.

#### Monthly Income Level

Figure 2C depicted the pandemic-induced changes in consumer purchase behavior according to monthly income levels. As can be seen, there was a significant variation. Individuals with no income or a monthly income of less than RMB 2,000 exhibited smaller change in their purchase object, place, timeframe, and method. People with a monthly income between RMB 5,001 and RMB 8,000 or above RMB 8,001 showed a greater change in their purchase object, place, and timeframe. In terms of purchase method, less variation was shown across monthly income levels. This is because the pandemic has prevented people from returning to work, resulting in a reduction in current or future household income, and because it has also affected people’s emotions and cognitions by instilling fear and anxiety about the future in them, prompting people to save preventively.

#### Education Attainment

Figure 2D showed the changes in consumer purchase behavior by education attainment. The changes in consumer purchase object and motive varied less across different education attainment levels compared to the changes in purchase place, timeframe, and method. Individuals with postgraduate or higher education attainment showed a small change in all aspects of purchase behavior. Unlike other demographic characteristics, education attainment had less of an impact on consumer purchase behavior.

### Regression Modeling

To examine the impact of consumer purchase behavior changes on the business model design of consumer services companies, this paper constructed a regression model as follows. As shown in equation (3), BMD represents business model design, which includes the novelty-centered business model design (NMB) and the efficiency-centered business model design (EBM). CPC is consumer purchase behavior changes, which includes the changes in purchase object (PO), the changes in purchase motive (PR), the changes in purchase place (PP), the changes in purchase timeframe (PT), and the changes in purchase method (PW). The relationships between BMDs and CPCs are examined using the following model:


$$B\,M\,D=\beta_{0}+\beta \,C\,P\,C+\beta_{1}\,A\,g\,e+\beta_{2}\,G\,e\,n\,d\,e\,r+\beta_{3}\,I\,n\,c\,o\,m\,e$$

(3) $$+\beta_{4}\,E\,d\,u+\varepsilon$$

Table 2 displayed the regression results for the relationship between the pandemic-induced changes in consumer purchase behavior and novelty-centered business model design. The regression result for Model 1 (M1) showed that the changes in consumer purchase object has a positive impact on the novelty-centered business model design (0.584, *p* < 0.001). The regression coefficient of the change in purchase object and novelty-centered business model was 0.584, and it was significantly positively correlated at the 1% level. That was, the greater the changes in the purchase object, the more inclined the consumer services companies is to design a novelty-centered business model. H1a is validated. Similarly, the results for Models 2–5 showed that the change in consumer purchase motive, place, timeframe, and method all have a positive impact on the novelty-centered business model design (0.583, *p* < 0.001; 0.516, *p* < 0.001; 0.505, *p* < 0.001; 0.459, *p* < 0.001, respectively). The regression coefficient of the change in purchase motive, place, timeframe, and method and novelty-centered business model was 0.583, 0.516, 0.505, 0.459, and it was significantly positively correlated at the 1% level. That was, the greater the changes in the purchase motive, place, timeframe, and method, the more inclined the consumer services companies is to design a novelty-centered business model. H1b, H1c, H1d, and H1e are validated. Model 6 integrated all parameters of consumer purchase behavior changes in order to test their combined impact on novelty-centered business model design. The results were consistent with Models 1–5, thus confirming the robustness of the findings. Therefore, consumer purchase behavior changes under the COVID-19 pandemic significantly contribute to the novelty-centered business model design of consumer services companies. Moreover, the variance inflation factor (VIF) of each model was less than 10. This indicated that multicollinearity in the models was not serious, and hence has no effect on the results.

**TABLE 2:** Consumer purchase behavior changes and novelty-centered business model design.

	NBM					
Variable	M1	M2	M3	M4	M5	M6
PO	0.584*** (0.021)					0.287*** (0.030)
PR		0.583*** (0.024)				0.220*** (0.030)
PP			0.516*** (0.024)			0.061* (0.030)
PT				0.505*** (0.024)		0.123*** (0.028)
PW					0.459*** (0.024)	0.157*** (0.025)
Age	−0.011 (0.072)	0.027 (0.075)	−0.151 (0.077)	−0.076 (0.078	0.073 (0.078)	−0.051 (0.069)
Gender	−0.082* (0.036)	0.011 (0.038)	0.056 (0.039)	−0.027 (0.039)	0.039 (0.040)	−0.033 (0.035)
Income	0.000 (0.007)	0.005 (0.008)	0.005 (0.008)	0.006 (0.008)	0.003 (0.008)	−0.002 (0.007)
Edu	−0.024 (0.027)	−0.050 (0.028)	−0.020 (0.028)	−0.046 (0.029)	−0.086** (0.029)	−0.042 (0.025)
Constant	0.338 (0.260)	0.226 (0.271)	0.739** (0.279)	0.599* (0.282)	0.206 (0.285)	0.551* (0.249)
Δ*R*^2^	0.317	0.259	0.221	0.204	0.180	0.386
*F*-test	162.575***	122.575***	99.628***	90.209***	77.208***	122.706***
VIF_*Max*_	2.140	2.135	2.136	2.135	2.140	2.455

***, **, * represent significant at the 1, 5, and 10% significant level, respectively; T-values are provided in parentheses.

Table 3 presented the regression results for the relationship between the pandemic-induced changes in consumer purchase behavior and the efficiency-centered business model design. Models 7–11 showed that the changes in purchase object, motive, place, timeframe, and method all have a significantly positive impact on the efficiency-centered business model design under COVID-19 (0.526, *p* < 0.001; 0.495, *p* < 0.001; 0.515, *p* < 0.001; 0.495, *p* < 0.001; 0.495, *p* < 0.001, respectively). H2a, H2b, H2c, H2d, and H2e are validated. Model 12 examined the combined effect of all parameters of consumer purchase behavior changes on efficiency-centered business model design. The results were consistent with Models 7–11, confirming the robustness of the results. Therefore, consumer purchase behavior changes over the course of the pandemic have a positive effect on the efficiency-centered business model design of consumer services companies. The variance inflation factor (VIF) of each model was less than 10. Again, this indicated that multicollinearity was not serious in the models, and hence had limited impact on the results.

**TABLE 3:** Consumer purchase behavior changes and efficiency-centered business model design.

	EBM					
Variable	M7	M8	M9	M10	M11	M12
PO	0.526*** (0.022)					0.228*** (0.031)
PR		0.495*** (0.025)				0.108** (0.031)
PP			0.515*** (0.023)			0.120*** (0.031)
PT				0.495*** (0.024)		0.127*** (0.029)
PW					0.495*** (0.023)	0.231*** (0.026)
Age	−0.025 (0.074)	0.012 (0.078)	−0.173* (0.077)	−0.096 (0.078)	0.050 (0.077)	−0.084 (0.071)
Gender	−0.134*** (0.038)	−0.050 (0.039)	−0.005 (0.039)	−0.088* (0.039)	−0.021 (0.039)	−0.076* (0.036)
Income	0.006 (0.008)	0.011 (0.008)	0.010 (0.008)	0.010 (0.008)	0.006 (0.008)	0.002 (0.007)
Edu	0.007 (0.028)	−0.016 (0.029)	0.014 (0.028)	−0.011 (0.029)	−0.053 (0.029)	−0.012 (0.026)
Constant	−0.057 (0.270)	0.168 (0.283)	0.377 (0.279)	0.226 (0.282)	−0.140 (0.279)	0.235 (0.255)
Δ*R*^2^	0.258	0.188	0.220	0.196	0.208	0.347
*F*-test	121.876***	81.455***	99.122***	86.036***	92.247***	103.833***
VIF_*Max*_	2.140	2.135	2.136	2.135	2.140	2.455

***, **, * represent significant at the 1, 5, and 10% significant level, respectively; T-values are provided in parentheses.

Based on the above evidence, consumer purchase behavior changes under the pandemic have a positive impact on both novelty- and efficiency-centered business model design. However, there is a significant variance in the magnitude of the coefficients, suggesting that consumer purchase behavior changes may have varying degrees of impact on each type of business model design. Specifically, the pandemic-induced changes in purchase object, motive, and method are more conducive to the novelty-centered business model design of consumer services companies (0.584 > 0.526; 0.593 > 0.495; 0.505 > 0.495, respectively); the changes in consumer purchase place have an equal effect on novelty- and efficiency-centered business model designs; and the changes in purchase method have a weaker impact on the novelty-business model design than the efficiency-centered business model design (0.459 < 0.495).

The changes in consumer purchase motive, object, and timeframe have a greater positive impact on the novelty-centered business model design. Consumers can retrieve much information on the internet to reduce their risks and uncertainty, thereby increasing trust in decision-making (Hussain et al., 2020). People are also more likely to purchase products or services recommended by others, and the internet is an effective way to obtain such information. As such, the changes in consumer purchase motive create an opportunity for consumer services companies to develop novelty-centered business models. During the pandemic, people have become more rational and quality-oriented in shopping, and consumer demand has shifted from quantity-focused to quality-and-quantity-focused. In this context, the market demands a wider range of products and services from companies, which can be achieved through novelty-centered business models. Therefore, the changes in purchase object have a positive impact on novelty-centered business model designs. In terms of purchase timeframe, when online shopping and home delivery cannot fulfill consumer demands in a timely manner due to pandemic disruptions and limited manpower, the consumer preference for community and near-home stores emerges. Therefore, the changes in consumer purchase timeframe have promoted novelty-centered business models, such as the physical community business model.

On the other hand, the changes in purchase method have predominantly favored efficiency-centered business models. The COVID-19 pandemic has put people at unprecedented risks. In order to reduce the risk, consumers have grown more interested in contactless delivery and mobile payment, which incorporate the omnichannel supply and provides the option to shop at any time. The customer need for low-risk, efficient, mobile, and fragmented shopping experiences opens up new business prospects for efficiency-centered business models. Therefore, the changes in consumer purchase method have a positive impact on efficiency-centered business model design for consumer services companies.

Finally, the changes in purchase place have a similar impact on novelty- and efficiency-centered business model design. Since COVID-19, there has been an increasing consumer demand for more diverse, personalized, convenient, and accessible shopping locations. Consumers want to shop in an innovative one-stop store that provides a safe or contactless environment, and this can be achieved by a business model that emphasizes both novelty and efficiency. Consumer services companies need to increase the diversity of their products and services while at the same time reducing their transaction costs to improve operational efficiency. Therefore, the changes in purchase place encourage both novelty- and efficiency-centered business model designs.

### Robustness Checks

#### Alternative Measures

In the previous section, we used the Item Response Theory (IRT) model to measure the variables related to consumer purchase behavior and business model design. To check the robustness of the results, we re-measured the variables using the weighted average method. The regression results were all significant, as shown in Table 4. All parameters of consumer purchase behavior changes have a significant impact on both novelty- and efficiency-centered business model design. The empirical results remain consistent with our prior findings. Therefore, our baseline results are robust.

**TABLE 4:** Alternative measures.

Variable	NBM					EBM				
	M13	M14	M15	M16	M17	M18	M19	M20	M21	M22
PO	0.726*** (0.028)					0.653*** (0.029)				
PR		0.614*** (0.025)					0.516*** (0.026)			
PP			0.544*** (0.026)					0.521*** (0.026)		
PT				0.509*** (0.023)					0.478*** (0.023)	
PW					0.478*** (0.024)					0.500*** (0.023)
Control	Yes	Yes	Yes	Yes	Yes	Yes	Yes	Yes	Yes	Yes
Constant	1.271*** (0.251)	1.561*** (0.254)	2.057*** (0.257)	1.918*** (0.254)	1.644*** (0.265)	1.865*** (0.258)	2.237*** (0.263)	2.511*** (0.257)	2.390*** (0.256)	1.943*** (0.260)
Δ*R*^2^	0.283	0.255	0.210	0.231	0.197	0.231	0.181	0.193	0.204	0.215
*F*-test	138.212***	119.940***	93.396***	105.452***	86.206***	105.431***	77.994***	84.349***	90.316***	96.482***
VIF_*Max*_	2.137	2.135	2.137	2.134	2.138	2.137	2.135	2.137	2.134	2.138

***, **, * represent significant at the 1, 5, and 10% significant level, respectively; T-values are provided in parentheses.

#### Control for Occupation

Another concern is the influence of missing variables on the relationship between consumer behavior changes and business model design. In the survey questionnaire, the respondents also provided information about their occupations. On the one hand, consumers’ occupation might alter their consumption behavior; while on the other hand, merchants might adjust their business strategies with respect to consumers with different occupations. As such, consumers’ occupation might affect the impact of consumer behavior changes on business model design, rendering the baseline results biased. Therefore, following Xue et al. (2019), we introduced respondents’ occupation into the baseline regressions and re-estimate the models. The results are displayed in Table 5. It shows that the results are highly consistent with baseline findings, with all regression coefficients being highly significant and positive (*p* < 0.01). Accordingly, our baseline results are again robust and reliable.

**TABLE 5:** Adding control variable (occupation).

Variable	NBM					EBM				
	M23	M24	M25	M26	M27	M28	M29	M30	M31	M32
PO	0.724*** (0.028)					0.652*** (0.029)				
PR		0.616*** (0.026)					0.517*** (0.027)			
PP			0.544*** (0.026)					0.521*** (0.026)		
PT				0.509*** (0.023)					0.477*** (0.023)	
PW					0.479*** (0.024)					0.500*** (0.023)
Control	Yes	Yes	Yes	Yes	Yes	Yes	Yes	Yes	Yes	Yes
Constant	1.288*** (0.293)	1.719*** (0.295)	2.149*** (0.301)	2.069*** (0.297)	1.834*** (0.308)	1.902*** (0.301)	2.401*** (0.307)	2.595*** (0.302)	2.548*** (0.300)	2.145*** (0.302)
Δ*R*^2^	0.283	0.256	0.210	0.231	0.198	0.229	0.180	0.191	0.203	0.214
*F*-test	63.345***	55.550***	43.080***	48.583***	40.118***	48.000***	35.842***	38.455***	41.241***	44.169***
VIF_*Max*_	5.377	5.382	5.377	5.383	5.378	5.377	5.382	5.377	5.383	5.378

***, **, * represent significant at the 1, 5, and 10% significant level, respectively; T-values are provided in parentheses.

## Conclusion

First of all, after the outbreak of the epidemic, there have been subtle changes in consumer buying groups. Male buying behavior has changed more. For example, they will increase the purchase of some necessities (Vázquez-Martínez et al., 2021). In the face of crisis, people’s utilitarian motivation is more significant (Voss et al., 2003). Therefore, people’s demand for daily necessities will increase substantially. In addition, the elderly no longer reject the purchase behavior through mobile methods, and many online shopping activities have been increased. This also makes life service companies need to further segment the market in terms of population in the future, such as adding more preferential activities for online service items for the elderly, so as to facilitate such people to further enhance their willingness to purchase.

Second, our findings suggest that changes in consumer purchase behavior have a significant positive impact on business model design. This influence reflects that consumers have put forward higher requirements for the marketing model of life service companies after experiencing the impact of the COVID-19 outbreak. According to the results of the study, changes in purchase object, purchase motive and purchase timeframe have a more profound impact on novelty-centered business model design. This shows that under the impact of the epidemic, consumer services companies should take rapid response measures, and carry out business model innovation according to the characteristics of the COVID-19 outbreak and changes in purchase behavior, such as: online transfer of sales model, expansion of target market, socialization and fragmentation of marketing model, unmanned retail, contactless service and enterprise platform integration.

Third, changes in purchase place and purchase method have a significant impact on efficiency-centered business model design. This shows that consumers currently hope that consumer services companies can reduce their selection costs, procurement costs and payment costs as much as possible, so as to ensure that they can obtain the required products or services more efficiently.

According to the research conclusions, this paper draws the following management implications: First, the consumer services companies based on new technologies should reduce their costs as much as possible and provide products or services efficiently. The company makes full use of the construction of new infrastructure such as Artificial Intelligence, Industrial Internet, and Internet of Things to power it, and makes innovations on this basis. In the future, the development direction of consumer services companies should be a deep and efficient combination of online and offline. In this way, a consumer-centric dynamic management model can be realized, and business models can be flexibly adjusted to respond to transform according to changes in the external environment. Second, enterprises should deeply explore consumers’ consumption preferences and stabilize the target market. The consumer market is unstable. While continuing to invest, companies should pay attention to the improvement of quality and service models, and deeply explore the consumption preferences of different consumers. On this basis, companies should continuously improve business models and stabilize the consumer market. Third, enterprises need to carefully introduce new models and services. During the outbreak of the epidemic, marketing models of live stream, community and short video have rapidly emerged. Not only have various e-commerce platforms started to adopt this marketing model, but some brand retailers have also begun to develop the live stream industry. According to the findings, consumers are enthusiastic about these emerging marketing models. At the same time, the unmanned retail model is also arousing the interest of consumers, and various intelligent retail products and services are put into operation, such as intelligent express cabinets, contactless distribution and unmanned convenience stores. The rapid development of these two types of models is affected by the epidemic environment, and managers should also consider the resources and capabilities of their own enterprise while rapidly innovating and introducing new models. At the same time, enterprises need to maintain a sense of crisis, cautiously introduce unfamiliar industries, and reasonably adopt various business models.

There are also limitations of this study that deserve future research attention. First, we explore the positive impact of consumer behavior changes on business model design in the consumer services sector. However, such relationship might vary across different sectors, cultures, and institution backgrounds. Future studies might examine it in a different research setting. Second, the picture of the nexus between consumer behavior changes and business model design might be incomplete. Future research might zoom into the consumption process or after-consumption behavior, investigating how the key findings might change with regard to different consumption stages.

## Data Availability Statement

The raw data supporting the conclusions of this article will be made available by the authors, without undue reservation.

## Author Contributions

HT collected literature. XS designed the research and wrote the manuscript. XS and HT performed the empirical analysis. XL provided the data. JT cleared data. DZ did the additional tests. All authors rewrote sections of the manuscript, contributed to manuscript revision, read, and approved the submitted version.

## Conflict of Interest

The authors declare that the research was conducted in the absence of any commercial or financial relationships that could be construed as a potential conflict of interest.

## Publisher’s Note

All claims expressed in this article are solely those of the authors and do not necessarily represent those of their affiliated organizations, or those of the publisher, the editors and the reviewers. Any product that may be evaluated in this article, or claim that may be made by its manufacturer, is not guaranteed or endorsed by the publisher.
